# Hepatic Stellate Cells Antagonize Sorafenib-Induced Ferroptosis in Hepatocellular Carcinoma by Upregulating the LINC00152/HSPB1 Axis

**DOI:** 10.3390/cancers18132106

**Published:** 2026-06-29

**Authors:** Yazhao Li, Jiayuan Yin, Rui Fan, Jiaojiao Su, Jiuhua Yi, Haoyu Wang, Bowen Yao

**Affiliations:** 1 Center for Translational Medicine, The First Affiliated Hospital of Xi’an Jiaotong University, Xi’an 710061, China; yazhaoli@xjtufh.edu.cn; 2 Department of Hepatobiliary Surgery, The First Affiliated Hospital of Xi’an Jiaotong University, Xi’an 710061, China; 3 Zonglian College, Xi’an Jiaotong University Health Science Center, Xi’an 710061, China; 4 Department of Cardiovascular Medicine, Kyoto University Hospital, Kyoto 606-8507, Japan

**Keywords:** hepatocellular carcinoma, ferroptosis, hepatic stellate cells, HSPB1, LncRNAs

## Abstract

**Background:** HCC remains one of the leading causes of cancer-related mortality worldwide, and the therapeutic efficacy of sorafenib is limited by the development of acquired resistance. Increasing evidence indicates that the tumor microenvironment, particularly HSCs, plays a pivotal role in modulating drug response; however, the underlying molecular mechanisms remain incompletely elucidated. **Methods:** Co-culture systems, mouse models, and biochemical assays were employed to evaluate the effects of HSCs on sorafenib sensitivity and ferroptosis in HCC cells. Transcriptomic analyses of data from The Cancer Genome Atlas were performed to identify key long non-coding RNAs (lncRNAs), followed by gain- and loss-of-function experiments to determine their biological roles. The underlying molecular mechanisms were further investigated through expression profiling, correlation analyses, and RNA stability assays. **Results:** HSCs markedly reduced the sensitivity of HCC cells to sorafenib by inhibiting ferroptosis, as evidenced by decreased levels of ferrous iron, reactive oxygen species, and lipid peroxidation, accompanied by increased glutathione content and activation of the *NRF2* signaling pathway. LINC00152 was identified as a critical lncRNA that was upregulated in both HCC tissues and HCC cells co-cultured with HSCs, and its high expression was associated with poor prognosis. Functional studies demonstrated that LINC00152 promoted sorafenib resistance and suppressed ferroptosis both in vitro and in vivo. Mechanistically, LINC00152 enhanced HSPB1 expression by stabilizing its mRNA. Notably, HSPB1 knockdown reversed the effects of LINC00152, restoring ferroptosis and drug sensitivity to sorafenib. **Conclusions:** These findings reveal a novel HSCs–LINC00152–HSPB1 axis that promotes ferroptosis resistance and sorafenib tolerance in HCC. Targeting this pathway may represent a promising therapeutic strategy for overcoming drug resistance and improving clinical outcomes in patients with HCC.

## Simple Summary

Sorafenib, a first-line targeted therapy for advanced hepatocellular carcinoma (HCC), functions as a potent inducer of ferroptosis, an iron-dependent form of regulated cell death. In this study, we observed that co-culture with hepatic stellate cells (HSCs) significantly suppressed sorafenib-induced ferroptosis in HCC cells. To investigate the underlying molecular mechanisms, we established an HSCs and HCC cell lines co-culture model to identify and validate the upstream and downstream regulatory pathways involved in this ferroptosis resistance. Through this model, we successfully identified the HSCs–LINC00152–HSPB1 axis as a novel signaling pathway that mediates ferroptosis inhibition, highlighting a potential therapeutic target for overcoming ferroptosis evasion in HCC.

## 1. Introduction

Hepatocellular carcinoma (HCC), the most common type of primary liver cancer, ranks as the seventh most frequently diagnosed malignancy worldwide and the second leading cause of cancer-related mortality [1]. Sorafenib, the first multikinase inhibitor approved for the treatment of advanced HCC, has provided a valuable therapeutic option for many patients. In recent years, the efficacy of combination targeted therapies for HCC has gained increasing recognition [2]. Initially, sorafenib was identified as an inhibitor of multiple oncogenic kinases; however, subsequent studies revealed that it also suppresses HCC progression through a distinct mechanism. Specifically, sorafenib inhibits system Xc^−^ activity independently of kinase targets or glutathione peroxidase (GPX) proteins, resulting in dysregulated iron metabolism and ultimately triggering a unique form of programmed cell death known as ferroptosis [3].

Ferroptosis is characterized by the accumulation of reactive oxygen species (ROS) and lipid peroxidation. Depletion of glutathione (GSH) further exacerbates ROS accumulation and increases the availability of free iron. Enhanced iron uptake coupled with impaired iron efflux leads to intracellular iron overload [4]. Iron is an essential trace element in the human body. Ferrous iron (Fe^2+^) is absorbed by duodenal epithelial cells through divalent metal transporter 1 (DMT1). Following release across the basolateral membrane via ferroportin (FPN), Fe^2+^ is oxidized to ferric iron (Fe^3+^) by the membrane-bound ferroxidase hephaestin (HEPH) and subsequently binds to transferrin (Tf) [5]. Most circulating iron exists as Tf–Fe^3+^ complexes and is taken up by cells through transferrin receptor 1 (TfR1)-mediated endocytosis. Cancer cells accumulate catalytically active free iron by upregulating iron uptake proteins (e.g., TfR1 and DMT1), downregulating iron efflux proteins (e.g., FPN), and reprogramming iron storage and release mechanisms, including ferritin turnover through NCOA4-mediated ferritinophagy. These adaptations support rapid proliferation, epithelial–mesenchymal transition, metastasis, and immune evasion [6,7,8,9]. In addition, mitochondrial ROS production is directly or indirectly regulated by lipid metabolic pathways involving *SLC7A11* and *GPX4*. Heat shock protein beta-1 (HSPB1) functions as a key negative regulator of ferroptosis by stabilizing the cytoskeleton, limiting iron uptake, and suppressing lipid ROS production through Heat shock factor 1 (HSF1)-dependent transcriptional activation and protein kinase C (PKC)-mediated phosphorylation [10].

Hepatic stellate cells (HSCs) are a specialized population of liver-specific fibroblasts that were originally referred to as lipid-storing cells. Activated HSCs secrete a variety of cytokines, including transforming growth factor-β1 (TGF-β1), epidermal growth factor, hepatocyte growth factor, and insulin-like growth factors (IGFs), which promote tumor growth, invasion, and metastasis. HSCs can modulate the phenotypic behavior of HCC cells through the activation of receptor-mediated signaling pathways, including the TGF-β1/Smad and CXCL12/CXCR4 axes [11,12]. However, whether HSCs influence iron metabolism in HCC and contribute to resistance against sorafenib-induced ferroptosis remains unknown.

Long non-coding RNAs (lncRNAs) constitute a large class of non-protein-coding transcripts that have emerged as important regulators of gene expression. Previous studies have shown that lncRNAs participate in diverse biological processes by modulating mRNA stability, interacting with microRNAs, regulating transcription, and influencing protein expression and function, thereby affecting downstream signaling pathways [13,14]. LINC00152 is a well-characterized lncRNA that is frequently upregulated in HCC and has been implicated in tumor cell proliferation, invasion, and metastasis [15,16,17]. Nevertheless, its role in ferroptosis-related tumor microenvironments remains poorly understood.

Our research team has been investigating the interactions between HSCs and HCC cells and has found that LINC00152 expression is markedly upregulated in HCC cells treated with HSC-conditioned medium or co-cultured with HSCs. Notably, ferroptosis was significantly attenuated under these conditions. Further experiments demonstrated that HSC-induced upregulation of LINC00152 promotes resistance to sorafenib-induced ferroptosis in HCC cells by stabilizing HSPB1 mRNA. In the present study, we established both in vitro and in vivo models through the co-culture or co-injection of HSCs and HCC cells to investigate the protective effects of HSCs on HCC cells. Specifically, we explored how activation of the LINC00152/HSPB1 axis contributes to resistance against sorafenib-induced ferroptosis.

## 2. Methods

### 2.1. Cell Culture and Co-Culture System

The HCC cell lines Hep3B (catalog no. CL-0106) and Huh7 (catalog no. CL-0120), as well as the hepatic stellate cell (HSC) line (catalog no. CL-0560), were purchased from Procell Life Science & Technology Co., Ltd. (Wuhan, China) and cultured under standard laboratory conditions. HCC cells were cultured in Dulbecco’s Modified Eagle Medium (AF29498406, HyClone, HyClone, Logan, UT, USA) supplemented with 10% fetal bovine serum (A3120802, Gibco, Waltham, MA, USA) and 1% penicillin–streptomycin solution (SV30010, HyClone, Logan, UT, USA). HSCs were cultured in astrocyte medium (1693, ScienCell, Carlsbad, CA, USA). All cells were maintained at 37 °C in a humidified incubator containing 5% carbon dioxide. Cells in good condition and at the logarithmic growth phase were used for all experiments. Huh7 and Hep3B cells (2 × 10^6^ cells/well in 2 mL of medium) were seeded into six-well plates. After cell attachment, HSCs (2 × 10^6^ cells/well in 2 mL of medium) were seeded into Transwell inserts fitted with 0.8-μm pore-size membranes and co-cultured with HCC cells for 24 h. To determine whether HSC-specific medium affected the growth of Hep3B and Huh7 cells, both cell lines were cultured in HSC-specific medium for 7 days. No changes in cellular morphology or phenotype were observed.

### 2.2. Western Blot Analysis

Cells were removed from the incubator, and the culture medium was discarded. The cells were immediately washed with ice-cold phosphate-buffered saline (PBS) and lysed using radioimmunoprecipitation assay lysis buffer (Beyotime, Shanghai, China) at 4 °C for 15 min to extract total protein. Protein concentrations were determined using a bicinchoninic acid protein assay kit (Pierce, Rockford, IL, USA). Protein samples were mixed with 5x loading buffer and denatured at 95 °C for 7 min in a boiling water bath. Equal amounts of protein were separated via 12% sodium dodecylsulfate–polyacrylamide gel electrophoresis and transferred onto polyvinylidene fluoride membranes (Millipore, Bedford, MA, USA). Membranes were blocked with 5% skim milk at room temperature for 2 hand then incubated overnight at 4 °C with the following primary antibodies: *NRF2* (Abcam, Cambridge, UK, 1:1000), HSPB1 (Proteintech, Rosemont, IL, USA, 1:1000), *HMOX1* (Abcam, Cambridge, UK,1:1000), *FTL* (Abcam, Cambridge, UK, 1:1000), and β-actin (Proteintech, Rosemont, IL, USA, 1:1000). After washing, membranes were incubated with horseradish peroxidase (HRP)-conjugated secondary antibodies (Bio-Tech, Minneapolis, MN, USA, 1:1000) at room temperaturefor 2 h. Protein bands were visualized using enhanced chemiluminescence reagent (Millipore, Bedford, MA, USA) and imaged with an Amersham™ Imager 680 (GE Healthcare Life Sciences, Beijing, China). Band intensities were quantified using ImageJ software (version 1.8.0, National Institutes of Health, Bethesda, MD, USA). Relative protein expression levels were calculated as the ratio of the integrated optical density of the target protein band to that of β-actin. All experiments were independently repeated at least three times.

### 2.3. Gene Manipulation and RNA Analysis

Huh7 or Hep3B cells (2 × 10^6^ cells/well in 2 mL of medium) were seeded into six-well plates. When cells reached approximately 60–70% confluence, transfection was performed according to the manufacturer’s instructions. For siRNA-mediated knockdown of LINC00152, X-tremeGENE siRNA Transfection Reagent (04476093001, Roche, Switzerland) was used at a reagent-to-siRNA ratio of 10:2. For LINC00152 overexpression, X-tremeGENE HP DNA Transfection Reagent was used at a reagent-to-DNA ratio of 3:1. Plasmid DNA was prepared at a concentration of 0.1–2.0 µg/µL in sterile water. Cells were transfected with either the siRNA/transfection reagent mixture or the plasmid DNA/transfection reagent mixture. The siRNA sequences are provided in the Supplementary Materials. Cells were harvested 24–48 h after transfection.

Total RNA was extracted using TRIzol reagent (Invitrogen, Carlsbad, CA, USA) and reverse-transcribed into complementary DNA using a reverse transcription kit (Thermo Fisher Scientific, Waltham, MA, USA). Quantitative real-time PCR (qRT-PCR) was performed according to standard protocols using primers listed in the Supplementary Materials. β-actin served as the internal control, and relative gene expression levels were calculated using the 2^−ΔΔCt^ method. RNA stability was evaluated by treating cells with actinomycin D (5 μg/mL; MedChemExpress, Monmouth Junction, NJ, USA), followed by time-course qRT-PCR analysis.

### 2.4. Ferroptosis-Related Assays

Ferroptosis-associated parameters, including intracellular Fe^2+^, GSH, malondialdehyde (MDA), and ROS, were measured using commercial assay kits (Abcam, Cambridge, UK; Sigma-Aldrich, St. Louis, MO, USA; and Beyotime, Shanghai, China) according to the manufacturers’ instructions. ROS levels were assessed via flow cytometry following DCFH-DA staining. Detailed experimental procedures are provided in the Supplementary Materials.

### 2.5. Animal Experiments

All animal experiments were approved by the Institutional Animal Care and Use Committee of the School of Medicine, Xi’an Jiaotong University. Three-week-old male nude mice were acclimatized in a specific pathogen-free facility for 1 week before subcutaneous inoculation with either Huh7 cells alone (4 × 10^6^ cells/100 μL PBS) or a mixture of Huh7 cells and HSCs (4 × 10^6^ and 2 × 10^6^ cells/100 μL PBS). The first experiment consisted of two groups: a control group (Huh7 only) and a co-inoculation group (Huh7 + HSCs). The second experiment consisted of four groups: control, sorafenib, LINC00152-overexpression, and LINC00152-overexpression + sorafenib. The latter two groups were inoculated with Huh7 cells stably overexpressing LINC00152 (4 × 10^6^ cells/100 μL PBS). Stable overexpression of LINC00152 was established as described in Section 2.3. Beginning on day 8 post-inoculation, when tumor volumes reached 80–100 mm^3^, mice in the sorafenib and combination-treatment groups received intraperitoneal injections of sorafenib (10 mg/kg/day) every other day. Mice in the remaining groups received an equivalent volume of saline. Tumor volume was calculated using the formula: length × width^2^ × π/6. Tumor volume and body weight were recorded every other day. All mice were euthanized on day 22.

### 2.6. Hematoxylin–Eosin Staining (HE) and Immunohistochemical Staining (IHC)

For IHC analysis, subcutaneous tumors derived from mice co-injected with Huh7 cells and HSCs were paraffin-embedded and sectioned. After deparaffinization in xylene, sections were rehydrated through a graded ethanol series. Antigen retrieval was performed by microwave heating in the retrieval buffer. Endogenous peroxidase activity was blocked with hydrogen peroxide, and nonspecific binding was prevented by incubation with goat serum. Sections were incubated overnight at 4 °C with an anti-α-SMA primary antibody (1:1500), followed by incubation with the corresponding secondary antibody at room temperature for 10 min. HRP was then applied for an additional 10 min. Immunoreactivity was visualized using the DAB chromogenic detection method. For H&E staining, paraffin sections underwent the same deparaffinization and rehydration procedures described above. Sections were stained with hematoxylin for several minutes, differentiated with acid alcohol, blued with ammonia water, and rinsed thoroughly under running water. Sections were then counterstained with eosin for 2–3 min, dehydrated through graded ethanol solutions, cleared in xylene, and mounted for microscopic examination.

### 2.7. Statistical Analysis

Data are presented as the mean ± standard deviation from at least three independent experiments. Statistical analyses were performed using Student’s *t*-test or one-way analysis of variance, as appropriate. A *p* value < 0.05 was considered statistically significant.

## 3. Results

### 3.1. HSCs Promote Sorafenib Resistance in HCC by Inhibiting Ferroptosis

**Figure 1:**
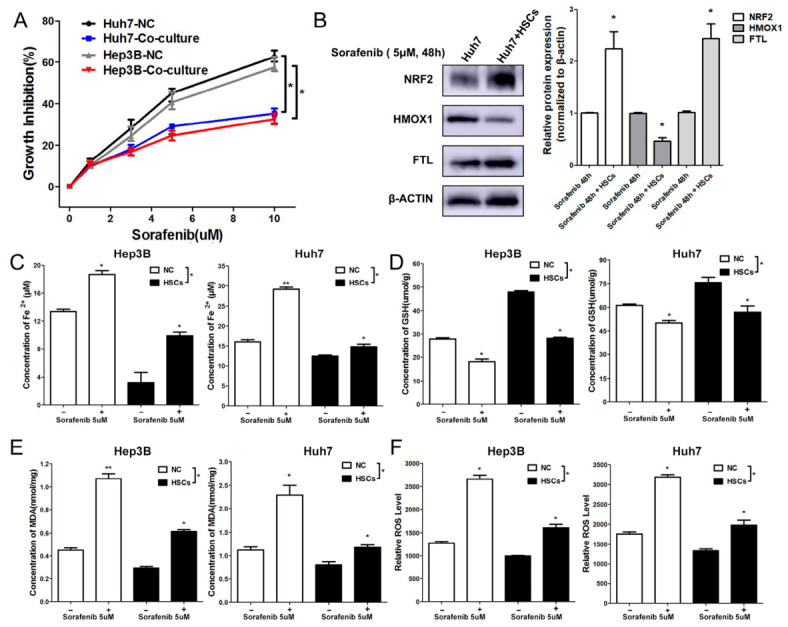
HSCs and HCC cell lines co-culture suppresses sorafenib-induced ferroptosis in HCC cells. (**A**) Growth inhibition curves of Huh7 and Hep3B cells cultured alone or co-cultured with HSCs after treatment with increasing concentrations of sorafenib (0–10 μM) for 48 h. Cell viability was normalized to the untreated control. Data are presented as mean ± SD (*n* = 3 independent experiments). (**B**) Western blot analysis of *NRF2*, *HMOX1*, and *FTL* expression in Huh7 cells with or without HSCs and HCC cell lines co-culture following 48 h of sorafenib treatment (5 μM). β-actin was used as a loading control. Representative blots from three independent experiments are shown. (**C**–**F**) Measurement of intracellular Fe^2+^ (**C**), GSH (**D**), MDA (**E**), and ROS (**F**) levels in Huh7 and Hep3B cells treated with 5 μM sorafenib under monoculture (−) or co-culture (+) conditions for 48 h. Data are presented as mean ± SD (*n* = 3 independent experiments). Asterisks indicate statistical significance: * *p* < 0.05, ** *p* < 0.01 (versus the respective control group).

To investigate the role of HSCs in the response to sorafenib, Huh7 and Hep3B cells were co-cultured with HSCs and treated with different concentrations of sorafenib. As shown in Figure 1A, compared with monoculture conditions, co-culture with HSCs significantly attenuated the growth-inhibitory effects of sorafenib on both Huh7 and Hep3B cells. At a sorafenib concentration of 5 μM, the difference in growth inhibition between the control and co-culture groups was most pronounced in both cell lines. Given that ferroptosis plays a key role in sorafenib-induced cell death, we subsequently examined the expression of the oxidative stress marker *NRF2* and the ferroptosis-related marker *HMOX1*. Western blot analysis revealed that co-culture significantly upregulated *NRF2* and its downstream target *FTL* (Figure 1B), indicating activation of the antioxidant defense pathway. In contrast, the ferroptosis-related marker *HMOX1* was slightly downregulated in the co-culture group compared with the monoculture group. To evaluate the effect of HSCs and HCC cell lines co-culture on sorafenib-induced ferroptosis, we measured the levels of Fe^2+^, ROS, GSH, and MDA in Huh7 and Hep3B cells. Compared with the sorafenib-only treatment group, intracellular Fe^2+^ and ROS levels were significantly reduced in the co-culture group (both *p* < 0.05), whereas GSH levels were significantly increased (*p* < 0.05). In addition, the accumulation of MDA, a lipid peroxidation end product, was significantly reduced (*p* < 0.05). These results indicate that HSC–HCC co-culture effectively inhibits sorafenib-induced ferroptosis and alleviates oxidative stress and lipid peroxidation.

**Figure 2:**
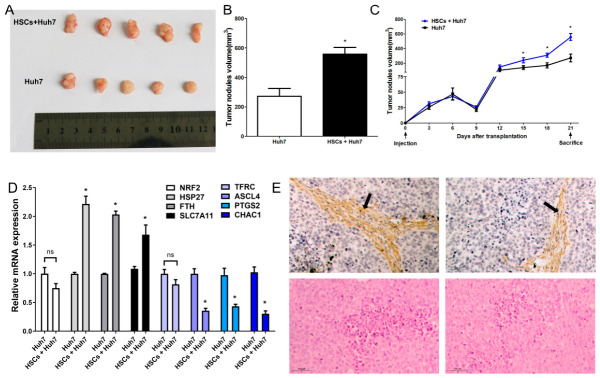
HSCs and HCC cell lines co-injection promotes tumor growth and alters ferroptosis-related gene expression in vivo. (**A**) Representative images of tumors derived from Huh7 cells alone (NC) or co-injected with HSCs in a subcutaneous xenograft mouse model. (**B**) Tumor weights at the endpoint (day 22). Data are presented as mean ± SD (*n* = 5 mice per group). * *p* < 0.05 vs. NC group (unpaired two-tailed Student’s *t*-test). (**C**) Tumor growth curves measured every other day from day 7 to day 21. Tumor volumes were calculated as (length × width^2^) × π/6. Data are presented as mean ± SD (*n* = 5 mice per group). * *p* < 0.05 vs. NC group (two-way ANOVA with repeated measures followed by Bonferroni’s post hoc test). (**D**) Relative mRNA expression levels of ferroptosis-related genes (*NRF2*, *TFRC*, *HSP27*, *ACSL4*, *FTH*, *PTGS2*, *SLC7A11*, and *CHAC1*) in tumor tissues measured via qRT-PCR. Expression was normalized to GAPDH and shown as fold change relative to the NC group. Data are presented as mean ± SD (*n* = 3 independent biological replicates per group). * *p* < 0.05 vs. NC group (unpaired two-tailed Student’s *t*-test). (**E**) Hematoxylin and eosin (H&E) staining and immunohistochemistry (IHC) analysis showing tumor histology and stromal characteristics associated with HSCs and HCC cell lines co-injection. Scale bars = 100 μm (or as appropriate). The arrows indicate the formation of HSC-associated stromal structures. Representative images from three independent tumors per group are shown.

To confirm these results in vivo, a nude mouse subcutaneous tumor model was established. Tumors derived from Huh7 cells co-injected with HSCs exhibited significantly enhanced growth compared with controls (Figure 2A–C). Furthermore, mRNA expression analysis revealed that ferroptosis resistance-related genes, including *SLC7A11*, *FTH*, and *HSP27*, were upregulated, while ferroptosis-promoting genes such as *ACSL4*, *PTGS2*, and *CHAC1* were downregulated in co-cultured tumors (Figure 2D). These findings suggest that HSCs may influence HCC progression by promoting ferroptosis resistance. Histological analysis further confirmed the formation of HSC-associated stromal structures (Figure 2E).

To validate these findings in vivo, we established a subcutaneous xenograft model in nude mice. Huh7 cells were injected subcutaneously either alone or together with HSCs. Compared with tumors derived from Huh7 cells alone, tumors generated by co-injection of Huh7 cells and HSCs exhibited significantly greater volume and weight (Figure 2A–C), indicating that HSCs promote HCC tumor growth in vivo. To further investigate the underlying molecular mechanisms, we examined the mRNA expression of ferroptosis-related genes in xenograft tissues. Compared with tumors derived from Huh7 cells alone, co-transplanted tumors exhibited significantly upregulated expression of anti-ferroptotic genes (*SLC7A11*, *FTH*, and *HSP27*) and significantly downregulated expression of pro-ferroptotic genes (*ACSL4*, *PTGS2*, and *CHAC1*) (Figure 2D). This gene expression profile was highly consistent with the findings from the in vitro co-culture experiments, further supporting the hypothesis that HSCs protect tumor cells by suppressing ferroptosis. Histological analysis further confirmed the formation of HSC-derived stromal structures in the co-grafted tumors (Figure 2E), suggesting that HSCs can successfully engraft in vivo, interact with tumor cells, and contribute to the establishment of a microenvironment conducive to tumor growth.

Collectively, these results demonstrate that HSCs enhance sorafenib resistance in HCC by suppressing ferroptosis.

### 3.2. LINC00152 Is Upregulated in HCC and Mediates HSC-Induced Ferroptosis Resistance

**Figure 3:**
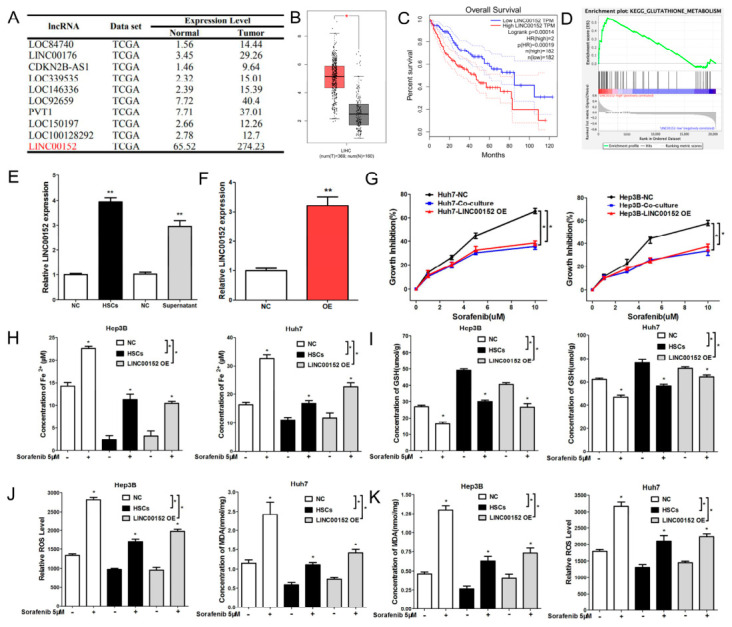
LINC00152 is upregulated in HCC and suppresses ferroptosis, reducing sorafenib sensitivity. (**A**) Differentially expressed lncRNAs identified from The Cancer Genome Atlas (TCGA) LIHC dataset, highlighting LINC00152 (red) among the top upregulated lncRNAs in tumor tissues compared with normal tissues (|log_2_FC| > 2, adjusted *p* < 0.05). (**B**) Expression levels of LINC00152 in 369 HCC tumor tissues versus 160 paired adjacent normal tissues from the TCGA-LIHC cohort. Data are presented as mean ± SD. * *p* < 0.05 (unpaired two-tailed Student’s *t*-test). (**C**) Kaplan–Meier overall survival analysis of HCC patients stratified by median LINC00152 expression (high vs. low). *p* value was determined via log-rank test (*n* = 364). The shaded areas between the doted lines of the same color denote the 95% confidence intervals. (**D**) KEGG pathway enrichment analysis of genes co-expressed with LINC00152 in the TCGA-LIHC dataset. Top enriched pathways related to tumor progression and metabolism are shown. (**E**) Relative LINC00152 expression in Huh7 and Hep3B cells, HSCs and HCC cell lines co-culture, and HSC-conditioned medium (CM) conditions for 48 h. Data are presented as mean ± SD (*n* = 3 independent experiments). ** *p* < 0.01 vs. control (one-way ANOVA followed by Tukey’s post hoc test). (**F**) Validation of LINC00152 overexpression efficiency. Huh7 cells were transfected with an empty vector (control) or LINC00152 overexpression plasmid. After 48 h, LINC00152 expression was measured via qRT-PCR and normalized to β-actin. Data are presented as mean ± SD (*n* = 3 independent experiments). ** *p* < 0.01 vs. control (unpaired two-tailed Student’s *t*-test). (**G**) Growth inhibition curves of Huh7 and Hep3B cells (negative control, co-culture, and LINC00152 overexpression) treated with increasing concentrations of sorafenib for 48 h. Cell viability was normalized to untreated control. Data are presented as mean ± SD (*n* = 3 independent experiments). * *p* < 0.05, * *p* < 0.05 for LINC00152 OE vs. control; * *p* < 0.05 for co-culture vs. control (two-way ANOVA with Bonferroni’s post hoc test). (**H**–**K**) Measurements of intracellular Fe^2+^ (**H**), GSH (**I**), ROS (**J**), and MDA (**K**) levels in Huh7 cells under control, LINC00152 overexpression, or co-culture conditions treated with 5 μM sorafenib for 48 h. Data are presented as mean ± SD (*n* = 3 independent experiments). * *p* < 0.05, * *p* < 0.05 vs. control (one-way ANOVA followed by Tukey’s post hoc test).

To identify key regulatory factors involved in HSC-mediated sorafenib resistance, we performed differential expression analysis of lncRNAs using the Cancer Genome Atlas (TCGA) Liver Hepatocellular Carcinoma (LIHC) dataset. Among the differentially expressed lncRNAs, LINC00152 was significantly upregulated in tumor tissues (Figure 3A), consistent with the increased expression observed in our previous co-culture experiments. Further analysis revealed that LINC00152 expression was significantly higher in HCC tissues than in matched adjacent non-tumor tissues (Figure 3B, *p* < 0.05). Kaplan–Meier survival analysis indicated that high LINC00152 expression was significantly associated with poorer overall survival in patients with HCC (Figure 3C, log-rank *p* < 0.05). Kyoto Encyclopedia of Genes and Genomics pathway enrichment analysis suggested that LINC00152 may participate in tumor progression and metabolic regulation (Figure 3D). To evaluate the impact of LINC00152 on postoperative recurrence risk, we conducted a disease-free survival (DFS) analysis using the TCGA-LIHC cohort. Kaplan–Meier curves demonstrated significantly poorer DFS in the high-expression group than in the low-expression group, with a clear divergence between the survival curves (log-rank *p* < 0.05). Furthermore, the median DFS was substantially shorter in the high-expression group (approximately 20 months) than in the low-expression group (approximately 30 months), and high LINC00152 expression was associated with an increased risk of recurrence (HR = 1.4, *p* < 0.05) (Supplementary Figure S1A). These findings suggest that elevated LINC00152 expression may serve as a predictive biomarker for early postoperative recurrence in patients with HCC.

In an in vitro co-culture system, LINC00152 expression was significantly increased in HCC cells following co-culture with HSCs or treatment with HSC-conditioned medium. Compared with monoculture conditions, LINC00152 expression was markedly elevated in HCC cells co-cultured with HSCs (Figure 3E, *p* < 0.05), suggesting that HSCs may induce LINC00152 upregulation in HCC cells. Simultaneously, enzyme-linked immunosorbent assay analysis of co-culture supernatants revealed elevated levels of TGF-β1 and tumor necrosis factor-α (TNF-α). However, the addition of neutralizing antibodies against transforming growth factor- β1 (TGF-β1) and TNF-α to the HSC–HCC co-culture system did not significantly alter LINC00152 expression in Huh7 cells (Supplementary Figure S1B), suggesting that other upstream regulators may be involved.

To investigate the role of LINC00152 in the response of HCC cells to sorafenib, we established Huh7 and Hep3B cell lines stably overexpressing LINC00152. qRT-PCR confirmed that LINC00152 expression was significantly increased in the overexpression groups compared with the empty-vector controls (approximately twofold, *p* < 0.01; Figure 3F). Based on these findings, we assessed cell growth inhibition following 48 h of treatment with different concentrations of sorafenib. As shown in Figure 3G, LINC00152 overexpression significantly attenuated sorafenib-induced growth inhibition in both Huh7 and Hep3B cells compared with the negative control group. These results suggest that LINC00152 contributes to sorafenib resistance. Consistent with this observation, LINC00152 overexpression significantly reduced intracellular Fe^2+^, ROS, and MDA levels while markedly increasing GSH levels (Figure 3H–K, *p* < 0.05), further supporting its role in suppressing ferroptosis.

**Figure 4:**
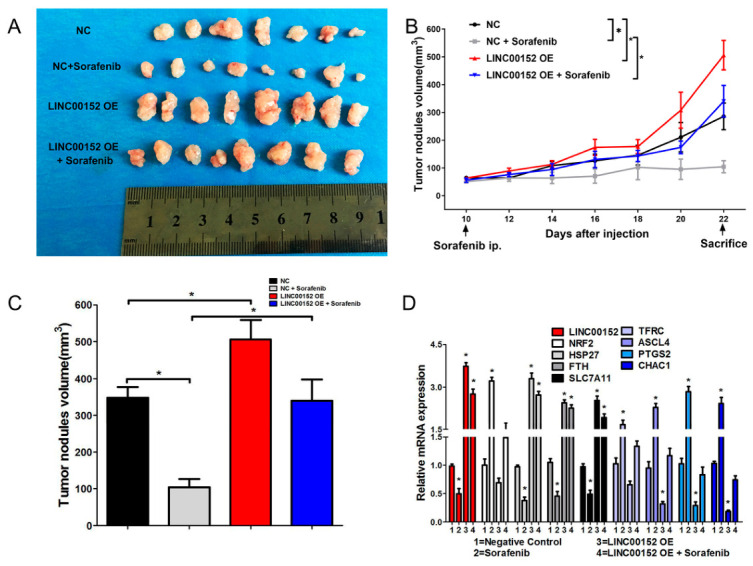
LINC00152 overexpression promotes tumor growth and attenuates the therapeutic effect of sorafenib in vivo. (**A**) Representative images of subcutaneous tumors from xenograft models with different treatments: NC (Huh7 alone), NC + sorafenib, LINC00152 OE (Huh7-LINC00152 alone), and LINC00152 OE + sorafenib. (**B**) Tumor growth curves measured every other day from day 7 to day 21. Tumor volumes were calculated as (length × width^2^) × π/6. Data are presented as mean ± SD (*n* = 5 mice per group). * *p* < 0.05 vs. NC group at the corresponding time point (two-way ANOVA with repeated measures followed by Bonferroni’s post hoc test). (**C**) Tumor weights at the endpoint (day 22). Data are presented as mean ± SD (*n* = 5 mice per group). * *p* < 0.05 vs. NC group (one-way ANOVA followed by Tukey’s post hoc test). (**D**) Relative mRNA expression levels of ferroptosis-related genes (*NRF2*, *TFRC*, *HSP27*, *ACSL4*, *FTH*, *PTGS2*, *SLC7A11*, and *CHAC1*) in tumor tissues measured via qRT-PCR. Expression was normalized to GAPDH and shown as fold change relative to the NC group. Data are presented as mean ± SD (*n* = 3 independent biological replicates per group). * *p* < 0.05 vs. NC group (one-way ANOVA followed by Tukey’s post hoc test).

In vivo experiments further validated these findings. LINC00152 overexpression significantly promoted tumor growth and attenuated the therapeutic efficacy of sorafenib (Figure 4A–C, *p* < 0.05). To assess the effect of LINC00152 on ferroptosis-related gene expression, we measured the mRNA levels of eight genes (*NRF2*, *TFRC*, *HSP27*, *ACSL4*, *FTH*, *PTGS2*, *SLC7A11*, and *CHAC1*) in tumor tissues via qRT-PCR (Figure 4D). Compared with the negative control group, the sorafenib monotherapy group showed significant upregulation of the ferroptosis marker genes *PTGS2* and *CHAC1* (both *p* < 0.05), as well as the ferroptosis-promoting gene *ACSL4* and the iron uptake-related gene *TFRC* (both *p* < 0.05). In contrast, the expression of the iron-storage gene *FTH* and the cystine transporter subunit *SLC7A11* was significantly reduced (all *p* < 0.05). Additionally, *NRF2* expression was significantly increased (*p* < 0.05), suggesting activation of an adaptive oxidative stress response. In the LINC00152 overexpression group, the expression of anti-ferroptotic genes (*FTH*, *SLC7A11*, and *HSP27*) showed an upward trend, whereas *NRF2* expression showed a downward trend, although these changes did not reach statistical significance. In the group receiving both LINC00152 overexpression and sorafenib treatment, *HSP27*, *FTH*, and *SLC7A11* expression levels remained significantly elevated compared with controls (all *p* < 0.05). However, the magnitude of upregulation was lower than that observed in the LINC00152 overexpression-only group. These findings suggest that LINC00152 may antagonize the anti-tumor effects of sorafenib by inhibiting ferroptosis.

### 3.3. LINC00152 Positively Regulates HSPB1 Expression

**Figure 5:**
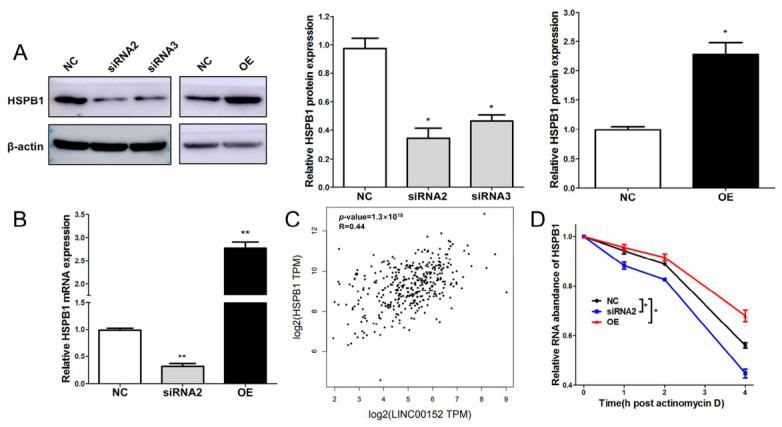
LINC00152 positively regulates HSPB1 expression at the post-transcriptional level by enhancing mRNA stability. (**A**) HSPB1 protein expression in Huh7 cells transfected with an empty vector (vector) or LINC00152 overexpression plasmid (LINC00152 OE). Representative blots from three independent experiments are shown. LINC00152 expression under knockdown and overexpression conditions to confirm transfection efficiency. Data are presented as mean ± SD (*n* = 3 independent experiments). * *p* < 0.05 vs. corresponding control (Student’s *t*-test). (**B**) HSPB1 mRNA expression under LINC00152 knockdown (si-LINC00152#1) and overexpression (LINC00152 OE) conditions in Huh7 cells. Data are presented as mean ± SD (*n* = 3 independent experiments). ** *p* < 0.01 vs. corresponding control (unpaired two-tailed Student’s *t*-test). (**C**) Correlation analysis between LINC00152 and HSPB1 mRNA expression. Spearman’s rank correlation coefficient (r = 0.44, *p* < 0.001) indicates a positive correlation. (**D**) RNA stability assay. Huh7 cells were transfected with si-NC, si-LINC00152#2, or LINC00152 OE plasmid. After 48 h, cells were treated with actinomycin D (5 μg/mL) to block new transcription. Total RNA was harvested at the indicated time points (0, 1, 2, 3, 4 h), and HSPB1 mRNA abundance was measured via qRT-PCR and normalized to the 0-h time point. Data are presented as mean ± SD (*n* = 3 independent experiments). * *p* < 0.05, * *p* < 0.05 for LINC00152 OE vs. si-NC; * *p* < 0.05 for si-LINC00152 vs. si-NC at the indicated time points (two-way ANOVA with Bonferroni’s post hoc test).

To further investigate the downstream molecular mechanisms by which LINC00152 regulates ferroptosis, we focused on HSPB1, a well-established negative regulator of ferroptosis. Through gain- and loss-of-function experiments, we found that LINC00152 positively regulates HSPB1 expression at both the mRNA and protein levels. Western blot analysis showed that LINC00152 overexpression in Huh7 cells significantly increased HSPB1 protein expression compared with the empty-vector control group (Figure 5A). qRT-PCR further confirmed that LINC00152 overexpression significantly increased HSPB1 mRNA levels in both Huh7 and Hep3B cells (*p* < 0.01; Figure 5B). Conversely, HSPB1 mRNA expression was significantly reduced following LINC00152 knockdown using specific siRNAs (*p* < 0.01; Figure 5B). Correlation analysis demonstrated a significant positive association between LINC00152 and HSPB1 expression in both the TCGA-LIHC dataset and our co-culture samples (Spearman’s r = 0.44, *p* < 0.001; Figure 5C). To further elucidate the mechanism by which LINC00152 regulates HSPB1, we performed RNA stability assays using actinomycin D to inhibit de novo transcription. The results showed that LINC00152 overexpression significantly prolonged the half-life of HSPB1 mRNA (*p* < 0.05), whereas LINC00152 knockdown accelerated its degradation (Figure 5D). These findings indicate that LINC00152 upregulates HSPB1 expression not by enhancing transcription initiation but by increasing the stability of HSPB1 mRNA at the post-transcriptional level.

### 3.4. LINC00152–HSPB1 Axis Mediates Ferroptosis Resistance

**Figure 6:**
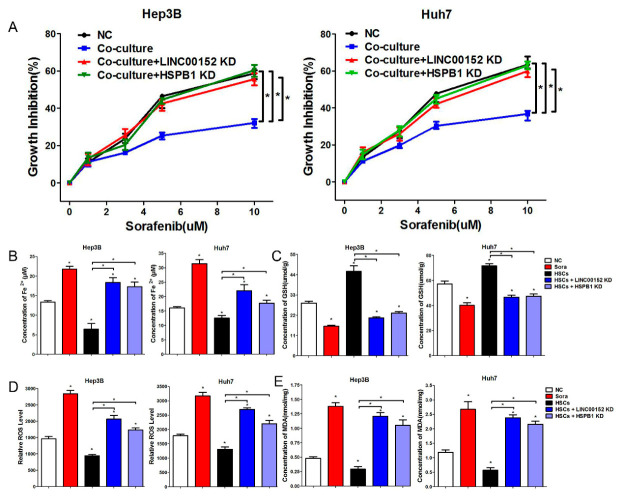
Knockdown of LINC00152 or HSPB1 restores ferroptosis sensitivity in HSCs and HCC cell lines co-culture. (**A**) Growth inhibition curves of Huh7 and Hep3B cells under different conditions: NC (monoculture), co-culture (HSCs and HCC cell lines co-culture), co-culture + si-LINC00152, and co-culture + si-HSPB1. Cells were treated with increasing concentrations of sorafenib for 48 h. Cell viability was normalized to the untreated control. Data are presented as mean ± SD (*n* = 3 independent experiments). * *p* < 0.05for co-culture + si-LINC00152 or co-culture + si-HSPB1 vs. co-culture alone (two-way ANOVA followed by Bonferroni’s post hoc test). (**B**–**E**) Quantification of intracellular Fe^2+^ (**B**), GSH (**C**), ROS (**D**), and MDA (**E**) levels in Huh7 and Hep3B cells under the indicated conditions (NC, co-culture, co-culture + si-LINC00152, co-culture + si-HSPB1) following treatment with 5 μM sorafenib for 48 h. Data are presented as mean ± SD (*n* = 3 independent experiments). * *p* < 0.05, vs. co-culture alone; * *p*< 0.05 vs. NC; * *p* < 0.05, HSCs vs. HSCs+LINC00152KD; * *p* < 0.05, HSCs vs. HSCs+HSPB1KD (one-way ANOVA followed by Tukey’s post hoc test).

To determine whether HSPB1 functions as a critical downstream effector of LINC00152-mediated ferroptosis inhibition, we performed rescue experiments. Under HSC–HCC co-culture conditions, knockdown of either LINC00152 or HSPB1 significantly attenuated the inhibitory effect of co-culture on sorafenib-induced ferroptosis, as evidenced by a further reduction in cell viability following sorafenib treatment (Figure 6A, *p* < 0.05 vs. co-culture control). Moreover, silencing LINC00152 or HSPB1 significantly increased intracellular Fe^2+^, ROS, and MDA levels while significantly decreasing GSH levels (Figure 6B–E, *p* < 0.05 vs. co-culture control), indicating restoration of ferroptotic sensitivity. Collectively, these results demonstrate that the LINC00152–HSPB1 axis is a key pathway mediating HSC-induced ferroptosis suppression and plays a critical role in the resistance of HCC cells to sorafenib-induced ferroptosis.

## 4. Discussion

In 2012, the team led by Brent R. Stockwell discovered that ferroptosis inducers such as erastin and sorafenib possess anticancer activity. Consequently, the effective dose of sorafenib required to induce ferroptosis is lower than the dose typically used for conventional targeted therapy (e.g., apoptosis induction). Through both in vitro and in vivo experiments, we demonstrated that sorafenib induces ferroptosis at a concentration of 5 μmol/L. At this concentration, HCC cells exhibited characteristic features of ferroptosis after 48 h of treatment. However, after 72 h, the cells underwent marked morphological changes, including partial detachment and cell death. Therefore, 48 h was uniformly selected as the treatment duration for sorafenib-induced ferroptosis throughout this study. In the in vivo experiments, administration of the same dose of sorafenib for 21 consecutive days did not result in any significant adverse effects on the behavior or general condition of the nude mice. It is noteworthy that under co-culture conditions, we observed increased *NRF2* protein expression accompanied by decreased *HMOX1* expression, indicating divergent expression patterns between these two proteins. This discrepancy from findings reported in some previous studies may be attributable to the dominant inhibitory effect of BACH1 and the complex regulation of *HMOX1* by multiple transcription factors. BACH1 is a potent transcriptional repressor of *HMOX1* that competes with *NRF2* for binding to antioxidant response elements within the *HMOX1* promoter. Importantly, the repressive effect of BACH1 is dominant; thus, inactivation of BACH1 is a prerequisite for *HMOX1* induction. Even when *NRF2* is activated, *HMOX1* transcription cannot be initiated if BACH1 remains bound to the promoter region [18]. Moreover, *HMOX1* expression is regulated by several transcription factors, including *NRF2*, HSF1, AP-1, and NF-κB [19]. Therefore, its ultimate expression level reflects the integrated activity of multiple signaling pathways. In addition, the in vivo transcriptomic data generated in this study were derived from a xenograft tumor model. As a result, tumor samples may have contained small amounts of RNA originating from host-derived stromal cells, which could have introduced background noise into the differential expression analysis. This limitation should therefore be considered when interpreting the in vivo transcriptomic findings.

HSCs play a crucial role in shaping the extracellular matrix within HCC lesions. Quiescent HSCs can be activated by hepatocellular carcinoma cells, and activated HSCs subsequently promote HCC cell proliferation and metastasis [20]. In addition to mediating fibrosis and increasing matrix stiffness [21], HSCs also participate in local immune regulation, thereby creating a microenvironment that supports tumor growth. Currently, no specific markers or inhibitors are available for selectively targeting activated HSCs. Extracellular vesicle uptake by HCC cells and cytokine receptor-mediated signaling are among the major mechanisms underlying HCC–HSC interactions. In the present study, LINC00152 expression was significantly upregulated following non-contact co-culture of HCC cells (Hep3B and Huh7 cell lines) with HSCs. Given that LINC00152 is already highly expressed in HCC, we speculate that this phenomenon may not primarily involve extracellular vesicle-mediated signaling. We further found that the levels of TGF-β1 and TNF-α in the culture supernatant were significantly elevated following co-culture of HSCs and HCC cells. Notably, simultaneous blockade of TGF-β1 and TNF-α signaling using neutralizing antibodies did not significantly alter LINC00152 expression in HCC cells. These findings suggest that although TGF-β1 and TNF-α are key mediators of HSC activation and liver fibrosis progression, they may not serve as the direct upstream regulators responsible for the transcriptional upregulation of LINC00152. Given the complexity of the bidirectional communication between HSCs and HCC cells, further elucidation of the upstream regulatory network governing LINC00152 expression is of considerable scientific interest. It represents an important direction for future research.

HSPB1, an ATP-independent molecular chaperone, promotes cellular survival under conditions of stress. Also known as heat shock protein 27 (*HSP27*), HSPB1 is highly expressed in a variety of human malignancies, including prostate cancer [22], ovarian cancer [23], gastric cancer [24], breast cancer [25] and lung cancer. In HCC, HSPB1 promotes invasion and metastasis through activation of the Akt signaling pathway while simultaneously preventing excessive iron accumulation and maintaining iron homeostasis. In the present study, we demonstrated that LINC00152 enhances HSPB1 protein expression by stabilizing HSPB1 mRNA, thereby counteracting sorafenib-induced ferroptosis in HCC cells. HSPB1 suppresses ferroptosis by reducing intracellular iron uptake and limiting the generation of lipid ROS. Its phosphorylated form, mediated by PKC, exerts this protective effect through the regulation of cytoskeleton-dependent iron uptake. [10] Further studies may establish HSPB1 as a valuable biomarker and therapeutic target in HCC. Future investigations should also determine whether HSPB1 plays a direct role in mediating HSC–HCC interactions.

## 5. Conclusions

In summary, our findings demonstrate that HSCs promote HCC progression by antagonizing sorafenib-induced ferroptosis through activation of the LINC00152/HSPB1 signaling axis. These results provide new mechanistic insights into the role of the tumor microenvironment in ferroptosis resistance and establish a theoretical foundation for the development of novel therapeutic strategies for HCC.

## Data Availability

The data analyzed in this study is subject to the following licenses/restrictions: The raw data supporting the conclusions of this article are available from the corresponding authors upon reasonable request. Requests to access these datasets should be directed to ybwsplendid@xjtufh.edu.cn.

## Supplementary Materials

The following supporting information can be downloaded at: https://www.mdpi.com/article/10.3390/cancers18132106/s1, Figure S1: LINC00152 is associated with poor prognosis and promotes tumor-supportive microenvironment via regulating HSPB1 and HSC-derived cytokines. Supplementary methods.
