# Supplementary material for: Hepatic Stellate Cells Antagonize Sorafenib-Induced Ferroptosis in Hepatocellular Carcinoma by Upregulating the LINC00152/HSPB1 Axis
Source: Cancers (Basel). 2026 Jun 29;18(13):2106. doi: 10.3390/cancers18132106 (PMC13359692; doi:10.3390/cancers18132106)
Supplement: Supplementary file 1 [file cancers-18-02106-s001.zip › cancers-4347486-supplementary.pdf]

Supplementary Figures

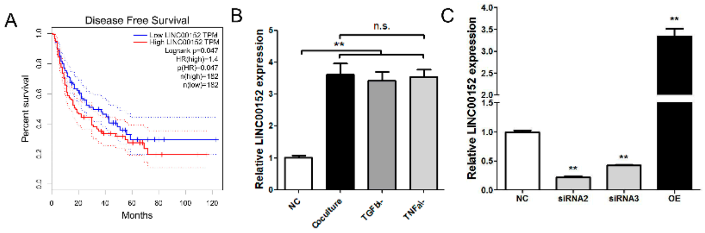

Supplementary Figure S1. LINC00152 is associated with poor prognosis and promotes tumor-supportive microenvironment via regulating HSPB1 and HSC-derived cytokines. (A) Kaplan–Meier curves of Disease-Free Survival (DFS) for 364 LIHC patients from the TCGA database. Patients were divided into high-expression (n = 182, red line) and low-expression (n = 182, blue line) groups based on the median LINC00152 expression level. The shaded areas between the doted lines of the same color denote the 95% confidence intervals. The log-rank test revealed that high LINC00152 expression was significantly associated with worse DFS ($p < 0.05$). (B) Cytokine expression in the supernatant of primary HSCs was assessed using ELISA. The results showed that, compared with HSCs cultured alone, levels of TGF-β1 and TNF-α in the HSCs supernatant were significantly elevated (**$p < 0.01$) following co-culture with HCC cells. The experiment was repeated three times. (C) HSPB1 protein expression in Huh7 cells transfected with control siRNA (si-NC) or two independent siRNAs targeting LINC00152 (si-LINC00152#1 and #2). Representative blots from three independent experiments are shown.

Supplementary Methods

Antibodies and Reagents

HSPB1 (18284-1-ap，Proteintech，Wuhan，China)

Nrf2 (ab62352，Abcam, Cambridge, MA, USA)

HSP27（18284-1-AP, Proteintech）

α-SMA（14395-1-AP，Proteintech）

β-actin (60008-1-Ig, Proteintech)

Sorafenib（28446-73-0，MedChemExpress）

RNA Interference and Gene Transfection

	sense	antisense
HSP27 siRNA	5'-ACGGUCAAGACCAAGGAUGdTdT-3'	5'-CAUCCUUGGUCUUGACCGUdTdT-3'
LINC00152 siRNA2	5’- UGACACACUUGAUCGAAUATT-3’	5’- UAUUCGAUCAAGUGUGUCATT-3’
LINC00152 siRNA3	5’- CCGGAAUGCAGCUGAAAGATT-3’	5’- UCUUUCAGCUGCAUUCCGGTT-3’

Overexpression transfection: Huh7 or Hep3B (4×10^6 per well in 2ml of medium) were seeded into six-well plates, after the cells adhere to the wall and fusion degree was about 70%-90%, follow the instructions took X-tremeGENE HP DNA Transfection Reagent (µl): DNA (µg) = 3:1. Prepareed a plasmid DNA solution with a concentration of 0.1-2.0 µg/µl in sterile water. Follow-up experiments were performed after transfection 24 hours. Overexpression sequence was purchased from (Genecopoeia Rockville, MD, USA).

LINC00152 Over Expression sequence：

ATGACAGACACCGAAAATCACGACTCAGCCCCCTCCAGCACCTCTACCTGTTGCCCGC
CGATCACAGCCGGAATGCAGCTGAAAGATTCCCTGGGGCCTGGTTCCAACCGCCCAC
TGTGGACTCTGAGGCCTCTGCATTTGCGGGTGGTCTGCCTGTAG

Quantitative Real-Time Polymerase Chain Reaction

The primers were described as following.

Primer	Forward	Reverse
NRF2	AGATTCACAGGCCTTTCTCG	CAGCTCTCCCTACCGTTGAG
β-actin	AGAGATGGCCACGGCTGCTT	ATTTGCGGTGGACGATGGAG
HSP27 FP	TCCCTGGATGTCAACCACTT	CAAAAGAACACACAGGTGGC
Nqo1	AGCGTTCGGTATTACGATCC	AGTACAATCAGGGCTCTTCTCG
Hmox1	AAGCCGAGAATGCTGAGTTCA	GCCGTGTAGATATGGTACAAGGA
Gsta1	CATTGAAGTGGTGAAGCACG	CTGGACTGTGAGCTGAGTGG
MT2	CAAACCGATCTCTCGTCGAT	AGGAGCAGCAGCTTTTCTTG
Slc7a11	TGGGTGGAACTGCTCGTAAT	AGGATGTAGCGTCCAAATGC
Gpx4	GATGGAGCCCATTCCTGAACC	CCCTGTACTTATCCAGGCAGA
ACSL4	CCTGAGGGGCTTGAAATTCAC	GTTGGTCTACTTGGAGGAACG

PTGS2	GGGAGTCTGGAACATTGGAA	GTGCACATTGTAAGTAGGTGGACT

Cell Viability Analysis

The cell viability was determined with the Cell Counting Kit-8 (CCK-8; Dojindo; Japan) assay. Huh7 or Hep3B (2×103 per well in 100 μL of medium) were seeded into 96-well plates, incubated in a 5% CO2, 37°C incubator, and treated with different measures for 24 hours, then added 10ul of CCK-8 reagent. Incubate the wells at 37°C for 1 hour, and measure the absorbance at 450 nm with a microplate reader. Calculation formula:

Cell survival rate=[(As-Ab)/(Ac-Ab)] ×100%, inhibition rate= [(Ac-As)/(Ac-Ab)] ×100%.

As: Absorbance of experimental well (including cells, culture medium, CCK-8 solution and drug solution)

Ac: Control well absorbance (containing cells, culture medium, CCK-8 solution, no drug solution)

Ab: Absorbance of blank hole (containing medium, CCK-8 solution, without cell and drug solution)

The measures of different experiment groups are as follow:

The first experiment: The experimentally treated liver cancer cells were divided into the NC group, the Sorafenib treatment group (Sorafenib supplementation concentrations were 0μM, 1μM, 3μM, 5μM, 10μM), and the hepatic stellate cell co-culture group (Sorafenib supplementation concentrations were respectively 0μM, 1μM, 3μM, 5μM, 10μM).

The second experiment: experimentally treated liver cancer cells were divided into NC group and LINC00152 overexpression group.

The third experiment: experimentally treated liver cancer cells were divided into NC group and HSPB1 siRNA silencing group.

Fourth experiment: The experimentally treated liver cancer cells were divided into NC group, co-cultured with hepatic stellate cells, LINC00152 knockdown group, and HSPB1 knockdown group.

Iron Assay

The relative iron concentration in cell lysates was assessed using the Iron Assay Kit (no. ab83366, Abcam). According to the kit instructions, washed the Hep3B or Huh7 with pre-cooled PBS to remove the residual medium, and then homogenize the cells with 4-10 times of the volume

iron detection buffer in ultrasound, centrifuge at 16,000 × g for 10 min. Next, we collected the supernatant to a clean tube, and add the sample (10 μL/well) to a 96-well plate, adjust the volume to 100 μL/well with iron detection buffer on ice. Fe2+ detection: Add 5 μL of detection buffer to each sample, incubated the plate at 37 ° C for 30 minutes. Then add 100 μL of iron probes to the sample wells, incubate the plate at 37°C in the dark for 60 minutes. Immediately put it on the colorimetric microplate reader to determine the absorbance (OD 593 nm). Calculate the concentration of Fe2+ in the sample.Iron Concentration= (Sa/ Sv) ×D

Note:

Sa = the iron content in the sample hole, calculated from the standard curve (nmol).

Sv = the sample volume (μL) added to the reaction well.

D = sample dilution factor. The molecular weight of iron ion is 55.845 g/mol

Lipid Peroxidation Assay

The relative malondialdehyde (MDA) concentration in cell lysates was assessed using a Lipid Peroxidation (MDA) Assay Kit (no. ab118970; Abcam). According to the manufacturer's instructions, we homogenized the cells (2×106 cells) with 303 μL of lysis buffer (buffer + BHT) on ice, then collected the supernatant by centrifuging the sample with 13,000 ×g for 10 minutes. Add 600 μL of TBA reagent and 200 μL of the supernatant to a new tube, incubate at 95°C for 60 minutes subsequently. Ice bath for 10 minutes and cool to room temperature. Finaly, 200 μL of the compound (containing MDA-TBA) were added to a well of 96-well plate to detect the absorbance (OD 532 nm) by microplate reader. Calculation formula:

MDA Concentration= [A/ (mg or ml)] × 4 × D=nmol/ml or nmol/mg

A=the amount of MDA in the sample, calculated from the standard curve (nmol). mg = initial tissue dosage (for example, 10 mg). mL = initial plasma dosage (0.020 mL).

4 = Take the correction factor of 200 μL from the 800 μL reaction mixture.

D = The sample dilution factor when the sample concentration is diluted to the range of the fitted standard curve (before setting the reaction well).

The experimental groups are the same as Iron Assay.

Glutathione Assay

The relative glutathione (GSH) concentration in cell lysates was assessed using the Glutathione Assay Kit (no. CS0260; Sigma). According to the manufacturer's instructions, we homogenized the tissue (100 mg) or the cells with 100 μL of 5% SSA solution, and centrifuged the sample at 12000 x g at 4ºC for 20 minutes. We collected the supernatant and kept it on ice, diluted the sample 5-20 times with glutathione buffer. After that, the diluted sample(20μL) , 100× mixed enzyme A (2μL) and reaction solution(80μL) were added to a well of 96-well plate, replenished the volume to 200μL/well with GSH buffer. Afterward, we detected absorbance (OD 450nm) of the sample by microplate reader. Calculation formula:

GSH amount in sample = (B/V*P) x D = nmol/mg

Note: B: is the amount of standard curve GSH (nmol),

V: is the sample volume (mL) added to the reaction well,

P: is the sample mg-protein concentration/Ml

D: is the sample dilution factor

The experimental groups are the same as Iron Assay.

Ros

Intracellular reactive oxygen species (ROS) levels were measured using the Reactive Oxygen Species Assay Kit (S0033S, Beyotime, China) with the fluorescent probe DCFH-DA. After treatment, cells were washed with PBS and incubated with 10 μM DCFH-DA (diluted 1:1000 in serum-free medium) at 37 °C for 20 min in the dark. Cells were then washed three times with serum-free medium to remove excess probe. ROS-positive controls were established using the ROS-inducing reagent Rosup provided with the kit, according to the manufacturer‘s instructions. Fluorescence intensity was immediately measured by flow cytometry (excitation 488 nm, emission 525 nm).
